# An integrative bioinformatics framework for functional annotation and prioritization of hypothetical proteins in *Bacillus thuringiensis* relevant to biological pest control

**DOI:** 10.1007/s42770-026-01985-x

**Published:** 2026-06-10

**Authors:** Vilmar Machado, Marcelo Rios Kwecko, Maria Alice Peglow Dos Reis, Jose Galian

**Affiliations:** 1 Federal Institute of Education, Science and Technology Sul-Rio-Grandense (IFSUL), Pelotas, Rio Grande do Sul Brazil; 2 Department of Zoology and Physical Anthropology, Faculty of Veterinary Medicine, University of Murcia, Murcia, Spain; 3 ArthropoTech S.L., Edificio Vitalis, Universidad de Murcia, 2a Planta, Despacho 2.15, Campus de Espinardo, 30100 Murcia, España

**Keywords:** *Bacillus thuringiensis*, Hypothetical proteins, Protein annotation, Functional inference, Bioinformatics, Computational biology

## Abstract

*Bacillus thuringiensis* is a widely used biological control agent whose genomes contain a substantial proportion of coding sequences annotated as hypothetical proteins, limiting functional interpretation and hindering their exploitation in biotechnology and genetic engineering. Here, we present an integrative and reproducible bioinformatics workflow for the systematic annotation and prioritization of hypothetical proteins from three *B. thuringiensis* serovars (Kurstaki, Pakistani, and Toumanoffi). The pipeline combines consensus-based functional annotation, virulence-associated prediction, subcellular localization analysis, pathogen-enrichment statistics, and structure-aware prioritization. Sequential filtering reduced an initial dataset of 2,052 hypothetical proteins to 11 non-redundant candidates supported by convergent computational evidence. Prioritized proteins included SGNH/GDSL hydrolases, iron–sulfur cluster repair proteins, HNH nucleases, transcriptional regulators, and envelope-associated proteins potentially related to stress adaptation and host-associated processes. Localization analyses identified extracellular, membrane-associated, and cytoplasmic candidates, suggesting participation in complementary adaptive functions. Structural prioritization based on physicochemical stability, topology-associated features, and docking-readiness criteria identified six proteins with favorable profiles for downstream structural and functional analyses. Rather than assigning definitive biological functions, this study provides a transferable computational framework for reducing the functional uncertainty associated with hypothetical proteins and supporting rational candidate selection for functional genomics and biotechnological applications in *Bacillus* and related bacterial systems.

**Supplementary Information:**

The online version contains supplementary material available at 10.1007/s42770-026-01985-x.

## Introduction

*Bacillus thuringiensis* (Bt) is a widely used biological control agent known for producing parasporal crystal (Cry and Cyt) proteins with insecticidal activity against a broad range of agricultural pests. Bt-based formulations account for most microbial biopesticides currently used worldwide, underscoring their relevance in sustainable pest management [1]. Beyond its classical role as a biopesticide, several *B. thuringiensis* strains have been reported to produce antibacterial, antifungal, antibiofilm, and surface-active compounds, as well as metabolites associated with plant growth promotion and environmental adaptation, highlighting its broader biotechnological potential [2]. The rapid expansion of genomic resources, with more than 700 *B. thuringiensis* genomes currently available in NCBI, provides an unprecedented opportunity to explore the genetic basis of these diverse traits.

Despite this extensive genomic information, a substantial proportion of predicted coding sequences, remain annotated as hypothetical proteins (HPs), lacking experimentally validated functional assignments. In bacterial systemsHPS can constitute a large fraction of the proteome and many have later been shown to participate in essential biological processes, highlighting the limitations of homology-based annotation approaches [3]. This persistent annotation gap represents a major challenge for functional genomics, comparative analyses, and and strain optimization, as uncharacterized proteins cannot be readily incorporated into functional models or exploited for targeted applications. At the same time, HPs represent a potentially valuable reservoir of of unexplored functional diversity and candidate targets for biotechnological applications contextual [4].

In *B. thuringiensis*, key virulence determinants such as Cry, Cyt, and Vip toxins are frequently associated with plasmid-borne elements, and genomic studies have revealed extensive accessory genomic diversity among strains [2, 5]. This genomic plasticity suggests that additional, poorly characterized proteins, including hHPs, may contribute to environmental adaptation, stress tolerance, and host-associated interactions through auxiliary or regulatory mechanisms. However, distinguishing biologically meaningful candidates from large pools of HPs remains challenging, particularly when relying on single-method predictions.

Integrative computational approaches combining conserved-domain detection, homology inference, structural information, and contextual biological analyses have emerged as valuable tools for improving functional annotation [6]. Previous studies have shown that combining sequence-based, structure-based, and physicochemical analyses significantly increases the reliability of hypothetical-protein characterization [3]. For example, SGNH/GDSL hydrolases have been associated with peptidoglycan remodeling and envelope stability [7], whereas iron–sulfur cluster repair proteins contribute to oxidative stress adaptation and redox homeostasis [8]. Similarly, disulfide oxidoreductases participate in protein folding and stabilization pathways associated with secreted or surface-exposed proteins [9–11]. Structural analyses have become particularly relevant in this context because protein topology and active-site organization can provide functional insights even when sequence similarity is limited [12].

Recent genome-mining studies have further highlighted the usefulness of integrative bioinformatics frameworks for identifying adaptive traits, virulence-associated factors, and novel functional elements in *B. thuringiensis* and related species [2, 13]. In this organism, auxiliary systems associated with stress response, envelope integrity, and regulatory control are likely to complement the activity of classical Cry/Cyt toxins and contribute to persistence across diverse ecological niches, including plant-associated and insect-associated environments [2, 14].

Here, we present a reproducible integrative bioinformatics workflow for the systematic annotation and prioritization of hypothetical proteins from three *B. thuringiensis* serovars (Kurstaki, Pakistani, and Toumanoffi). We hypothesize that (i) integration of complementary annotation strategies can reduce the functional uncertainty associated with hypothetical proteins more effectively than single-method approaches, (ii) subsets of these proteins contain convergent computational signals associated with adaptive and host-related processes, and (iii) incorporation of physicochemical and structural criteria enables the identification of candidates with increased suitability for downstream structural and functional analyses. To test these hypotheses, the proposed workflow combines consensus-based annotation, virulence-associated prediction, pathogen-enrichment analysis, subcellular localization profiling, and structure-aware prioritization to identify a refined set of biologically plausible candidates for future experimental validation.

## Materials and methods

### Genomic data retrieval and selection of hypothetical proteins

**Fig. 1:**
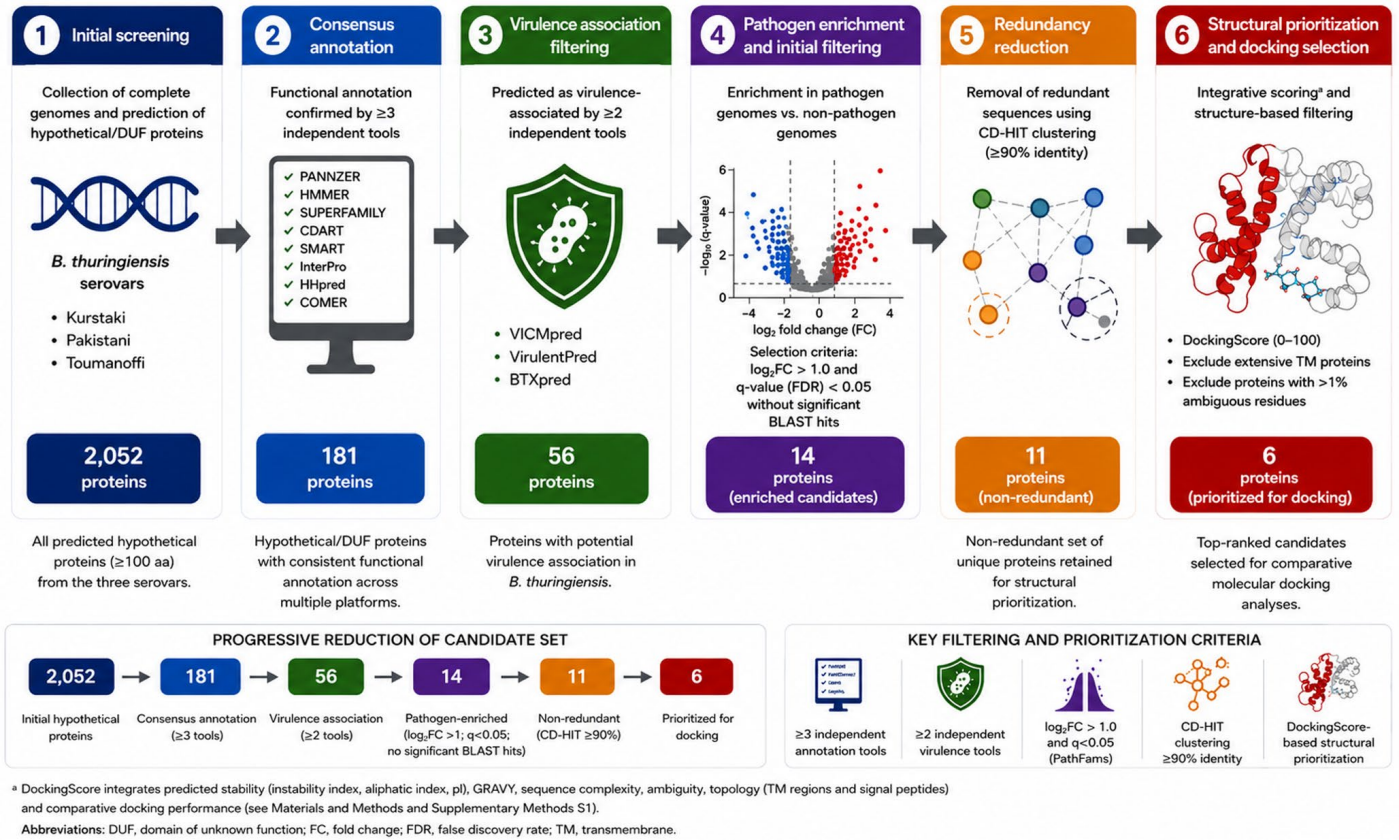
Integrative workflow for the screening, enrichment, and prioritization of hypothetical proteins in *Bacillus thuringiensis* serovars. Sequential filtering included consensus-based functional annotation, virulence-associated prediction, pathogen-enrichment analysis, redundancy reduction, and structure-aware prioritization. Progressive reduction of the candidate dataset is shown across analytical stages, from the initial set of 2,052 hypothetical proteins to six structurally prioritized candidates selected for comparative docking analyses. Selection thresholds and prioritization criteria applied at each stage are indicated within the workflow

Genome assemblies of *Bacillus thuringiensis* serovars Kurstaki, Pakistani, and Toumanoffi were retrieved from the NCBI database following Blackburn et al. [15]. Coding sequences annotated as hypothetical proteins (HPs) or containing domains of unknown function (DUFs) were extracted for downstream analyses. Only complete sequences ≥ 100 amino acids were retained to reduce the inclusion of truncated proteins and low-confidence open reading frames commonly observed in draft genome annotations [16]. The overall analytical workflow employed for annotation, enrichment analysis, redundancy reduction, and structural prioritization is summarized in Fig. 1.

Genome assemblies from three *Bacillus thuringiensis* serovars (Kurstaki, Pakistani, and Toumanoffi) were retrieved from the NCBI database following the selection criteria described by Blackburn et al. [15]. Protein-coding sequences annotated as hypothetical proteins (HPs) or containing domains of unknown function (DUFs) were extracted for downstream analyses. Only complete protein sequences ≥ 100 amino acids were retained to minimize the inclusion of truncated or low-confidence predictions. The overall analytical workflow is summarized in Fig. 1.

### Consensus-based functional annotation

Initial functional annotation was performed using PANNZER [17]. Functional predictions were subsequently validated using complementary annotation platforms, including HMMER [18], SUPERFAMILY [19], CDART [20], SMART [21, 22], InterPro [23], HHpred, and COMER. Only proteins supported by at least three independent tools were retained to improve annotation reliability and reduce false-positive assignments [3, 6].

Consensus-supported annotations and domain predictions are summarized in Supplementary Table S3.

### Virulence-associated prediction and pathogen-enrichment analysis

Virulence-associated predictions were performed using VICMpred [24], VirulentPred [25], and BTXpred [26]. Proteins predicted as virulence-associated by at least two tools were retained for further analysis.

Pathogen enrichment was evaluated using PathFams [27], comparing candidate proteins against pathogen versus non-pathogen genome groups. Proteins with log₂ fold change > 1.0 and FDR-adjusted Q-values < 0.05 were considered significantly enriched. Borderline candidates not meeting both thresholds were retained only when supported by complementary evidence, including consensus annotation, localization patterns, and structural prioritization criteria.

Virulence prediction outputs and enrichment metrics are summarized in Supplementary Table S4.

### Homology assessment and redundancy reduction

Candidate proteins were compared against the NCBI non-redundant (nr) and UniProt databases using BLASTp [28]. Sequences showing ≥ 90% identity to previously characterized proteins were excluded to prioritize poorly characterized candidates.

Redundancy reduction across serovars was performed using CD-HIT clustering [29] at ≥ 90% global sequence identity, retaining one representative sequence per cluster for subsequent analyses.

Representative similarity results and clustering outputs are summarized in Supplementary Table S2.

### Subcellular localization and topology prediction

Subcellular localization was inferred using a consensus strategy combining BUSCA [30], CELLO 2.0 [31], PSLpred [32], Phobius [33], and PSORTb v3.0 [34]. Proteins were classified as extracellular, membrane-associated, or cytoplasmic according to agreement among prediction tools.

Transmembrane helices were predicted using TMHMM v2.0 [35] and DeepTMHMM [36], while signal peptides were identified using SignalP 6.0 [37]. CCTOP [38] was additionally used to support topology prediction.

### Physicochemical characterization and structural prioritization

Physicochemical properties were calculated using ProtParam [39, 40], including molecular weight, theoretical isoelectric point (pI), instability index [41], aliphatic index, and GRAVY hydropathy values [42]. Additional sequence-quality criteria included prediction of transmembrane helices, signal peptides, low-complexity regions, and ambiguous residues.

Structural prioritization was performed using an integrative heuristic score (DockingScore; range 0–100) combining physicochemical stability, hydropathy, sequence complexity, and topology-associated features. Proteins containing extensive transmembrane regions or > 1% ambiguous residues were excluded from docking prioritization. Candidates with higher DockingScore values were considered more suitable for downstream structural analyses and comparative docking.

Detailed prioritization criteria, parameter ranges, and exclusion rules are summarized in Supplementary Table S1.

### Comparative molecular docking analyses

Protein structures were obtained from the AlphaFold Protein Structure Database or predicted using AlphaFold2. Regions with low structural confidence were excluded based on predicted local distance difference test (pLDDT) scores.

Potential ligand-binding pockets were identified using CASTp [43] and fpocket [44]. Candidate ligands were selected from the PubChem database according to predicted functional classes and conserved-domain information.

Protein and ligand structures were prepared using AutoDock Tools [45], and molecular docking simulations were performed using AutoDock Vina [46] with a rigid-receptor approach (exhaustiveness = 8). Docking analyses were conducted in duplicate, and binding energies were interpreted comparatively to evaluate relative interaction plausibility rather than definitive biochemical affinity.

### Reproducibility and computational implementation

All computational analyses were independently repeated to ensure consistency across tools and prediction runs. The workflow was designed as a modular and transferable framework integrating complementary computational evidence for hypothesis generation and candidate prioritization rather than definitive functional assignment.

## Results

### Identification and filtering of hypothetical proteins

A total of 2,052 hypothetical proteins (HPs) and domain-of-unknown-function (DUF)-containing proteins were initially identified across the three *Bacillus thuringiensis* serovars analyzed, including 593 from Toumanoffi, 767 from Pakistani, and 692 from Kurstaki (Table 1). Sequential application of consensus-based annotation, virulence-associated prediction, pathogen-enrichment analysis, redundancy reduction, and structural prioritization progressively refined the dataset into a restricted set of biologically plausible candidates.

**Table 1:** Sequential filtering and prioritization workflow applied to hypothetical proteins identified in *Bacillus thuringiensis* serovars. Sequential filtering integrated consensus-based functional annotation, virulence-associated prediction, pathogen-enrichment analysis, redundancy reduction, and structural prioritization. Consensus annotations were retained only when supported by at least three independent annotation tools, whereas virulence-associated candidates required agreement between at least two prediction platforms. Pathogen-enriched proteins correspond to candidates satisfying enrichment thresholds defined by PathFams analysis (log₂FC >1.0 and FDR-adjusted q <0.05). Redundancy reduction was performed using CD-HIT clustering at ≥90% sequence identity. Structurally prioritized proteins correspond to the final candidate set retained for downstream docking analyses

Serovar	Total proteins	Hypothetical proteins	Consensus annotations (≥ 3 tools)	Virulence-associated candidates (≥ 2 tools)	Pathogen-enriched candidates	Non-redundant proteins (CD-HIT ≥ 90%)	Structurally prioritized proteins
Toumanoffi	6303	593	48	19	5	4	3
Pakistani	6567	767	99	17	5	4	2
Kurstaki	6574	692	34	20	4	3	1
Total	**19**,**444**	**2**,**052**	**181**	**56**	**14**	**11**	**6**

Initial functional annotation using PANNZER and complementary tools identified 181 proteins with consensus-supported annotations. Subsequent virulence-associated prediction retained 56 candidates supported by at least two independent prediction platforms. Application of pathogen-enrichment criteria further reduced the dataset to 14 proteins significantly enriched in pathogen-associated groups. Redundancy reduction using CD-HIT clustering at 90% sequence identity generated a final set of 11 non-redundant protein entities, representing less than 1% of the original HP dataset.

Pakistani exhibited the highest number of consensus-supported annotations (99 proteins), whereas Kurstaki and Toumanoffi presented lower numbers of computationally tractable candidates, indicating variability in the proportion of annotatable HPs among serovars. The overall workflow and filtering strategy are summarized in Fig. 1, while quantitative reduction across analytical stages is presented in Table 1.

### Functional annotation and domain signatures

Consensus-based annotation revealed substantial functional diversity among the prioritized candidates, including hydrolases, oxidoreductases, nucleases, transcriptional regulators, toxin-immunity proteins, and envelope-associated factors (Table 2). Functional predictions derived from HHPred, COMER, InterPro, SUPERFAMILY, and Pfam databases showed strong agreement for multiple candidates, supporting the robustness of the annotation strategy.

**Table 2:** Pathogen-enriched hypothetical proteins prioritized after integrative annotation and enrichment analysis in *Bacillus thuringiensis* serovars. The table summarizes pathogen-enriched hypothetical proteins retained after consensus annotation, virulence-associated prediction, enrichment analysis, and redundancy reduction. Localization profiles correspond to consensus predictions obtained from multiple subcellular-localization tools. Structural-priority classification was determined according to integrative DockingScore evaluation and topology-associated filtering criteria

Protein ID	Serovar	Predicted functional class	Localization	log₂FC	FDR-adjusted q-value	Structural priority
WP_001091101.1	Toumanoffi	Histone acetyltransferase HPA2 (DUF1104)	Extracellular	5.02	3.52 × 10⁻¹¹	High
MCR6857976.1	Toumanoffi	SGNH/GDSL hydrolase	Extracellular	11.63	3.52 × 10⁻¹¹	High
MCR6868317.1	Toumanoffi	Iron–sulfur repair protein	Cytoplasmic	14.02	3.52 × 10⁻¹¹	High
MCR6823244.1	Pakistani	CpXC domain protein (Redoxin)	Cytoplasmic	3.93	2.69 × 10⁻⁶	High
MCR6861676.1	Toumanoffi	HNH nuclease protein	Cytoplasmic	4.98	3.06 × 10⁻⁶	High
MCR6823109.1	Pakistani	Immunity protein 49 (PTS)	Cytoplasmic	5.18	6.20 × 10⁻⁶	Moderate
…	…	…	…	…	…	…

SGNH/GDSL hydrolases represented one of the most recurrent functional classes identified across the analyzed serovars and consistently displayed strong pathogen-enrichment support. Iron–sulfur cluster repair proteins also exhibited elevated fold-change values and highly significant Q-values, suggesting potential association with oxidative stress adaptation and redox homeostasis. Additional recurrent functional signatures included HNH nucleases, helix–turn–helix (HTH) transcriptional regulators, DoxX-like proteins, HAAS-domain proteins, reductase/disulfide isomerases, and Cpx-associated envelope stress proteins.

Collectively, these annotations indicate enrichment of proteins potentially associated with adaptive, regulatory, and stress-response pathways. Consensus-supported functional annotations are summarized in Table 2, whereas detailed multi-platform annotation outputs are presented in Supplementary Table S3.

### Subcellular localization patterns

**Fig. 2:**
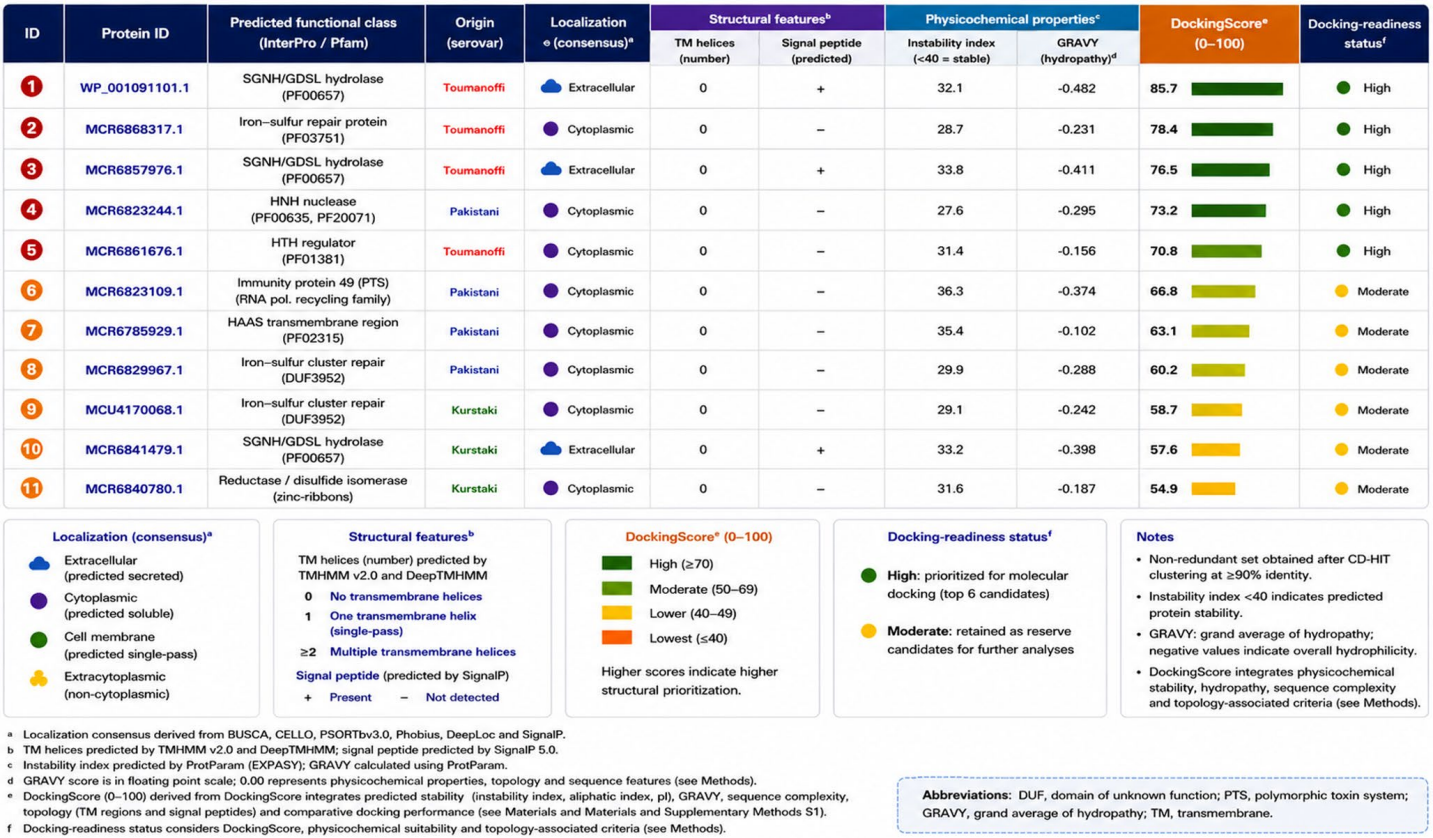
Structural and topological characterization of the 11 non-redundant hypothetical proteins prioritized for structural analysis in *Bacillus thuringiensis* serovars. Candidates retained after redundancy reduction (CD-HIT, ≥90% identity) were comparatively evaluated according to predicted functional class, subcellular localization, transmembrane topology, signal peptide occurrence, physicochemical stability, hydropathy, and integrative DockingScore prioritization. Consensus localization was inferred using multiple prediction tools, whereas topology-associated features included transmembrane helices and secretion signals. DockingScore values integrate physicochemical stability, hydropathy, sequence complexity, and topology-associated criteria to estimate structural suitability for downstream analyses. Candidates classified as “High” were prioritized for comparative molecular docking analyses

Consensus-based localization analyses distributed the prioritized proteins across cytoplasmic, membrane-associated, and extracellular compartments (Fig. 2). Cytoplasmic proteins predominated among the retained candidates and included HNH nucleases, HTH transcriptional regulators, histone acetyltransferase-associated proteins, and oxidative stress-related proteins. Membrane-associated candidates included HAAS-domain proteins, DoxX-like proteins, reductase/disulfide isomerases, and envelope-associated factors, whereas extracellular predictions were primarily associated with SGNH/GDSL hydrolases.

Most prioritized proteins lacked transmembrane helices, whereas extracellular candidates frequently presented signal peptides compatible with secretion-associated profiles.

Integration of multiple localization and topology-prediction tools generated internally consistent compartmentalization patterns supporting potential involvement in both intracellular adaptive processes and extracellular or envelope-associated functions.

Consensus localization profiles, signal peptide predictions, transmembrane topology, and structural-prioritization features are summarized in Fig. 2.

### Statistical enrichment and prioritization

**Fig. 3:**
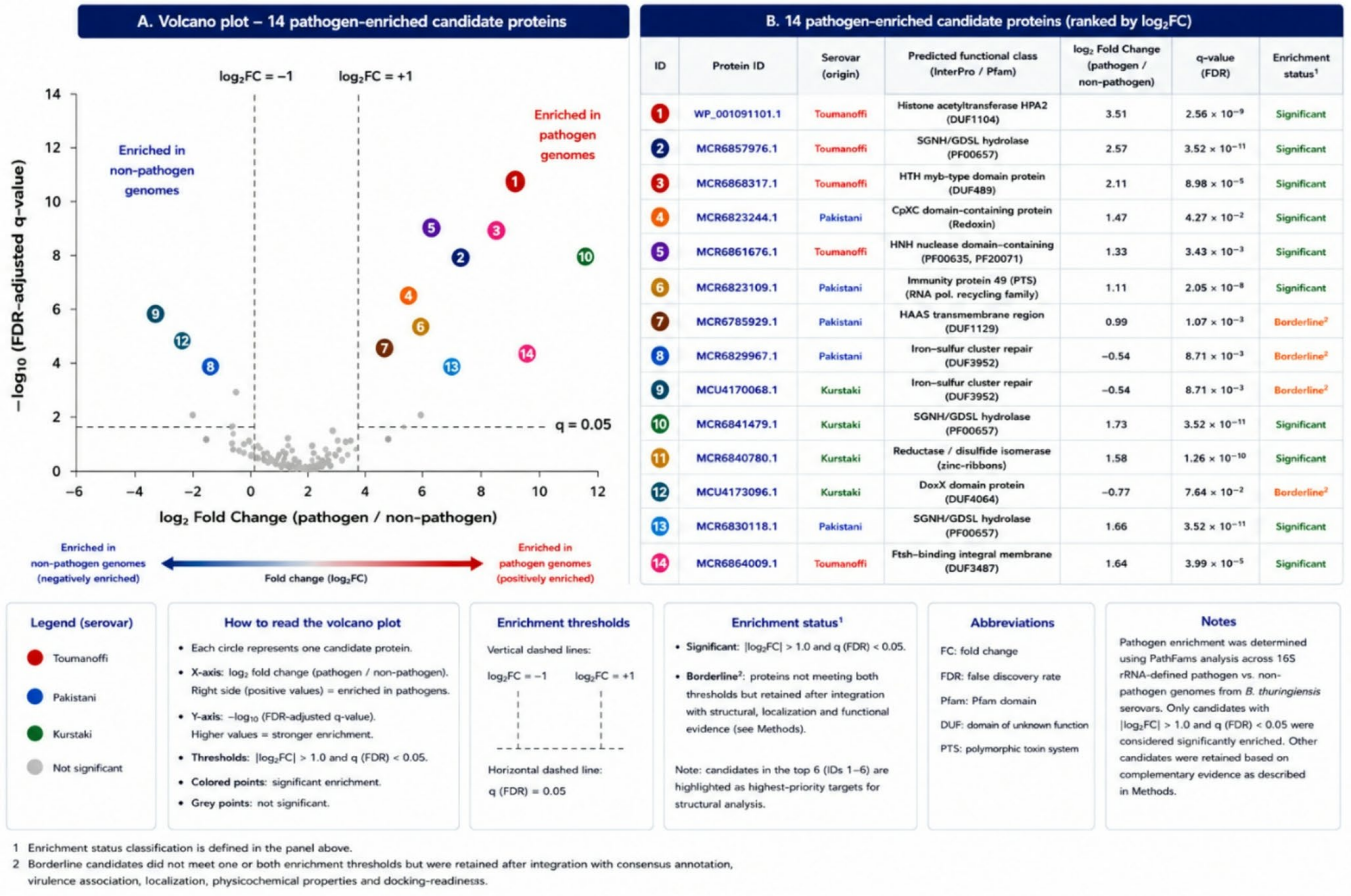
Pathogen-enrichment analysis of hypothetical proteins across *Bacillus thuringiensis* serovars. **A** Volcano plot showing the distribution of candidate proteins according to pathogen-enrichment fold change (log₂FC; pathogen vs. non-pathogen genomes) and statistical significance, (− log₁₀ FDR-adjusted q-values). Vertical dashed lines indicate enrichment thresholds (log₂FC = ± 1), and the horizontal dashed line indicates the significance threshold, (q = 0.05). Colored points represent prioritized candidate proteins retained after integrative filtering. **B** Ranked list of the 14 pathogen-enriched candidate proteins identified by PathFams analysis, including predicted functional classes, serovar origin, enrichment values, and statistical support. Proteins classified as “Borderline” did not satisfy all enrichment thresholds but were retained based on complementary evidence derived from consensus annotation, localization profiles, physicochemical properties, and structural prioritization

**Fig. 4:**
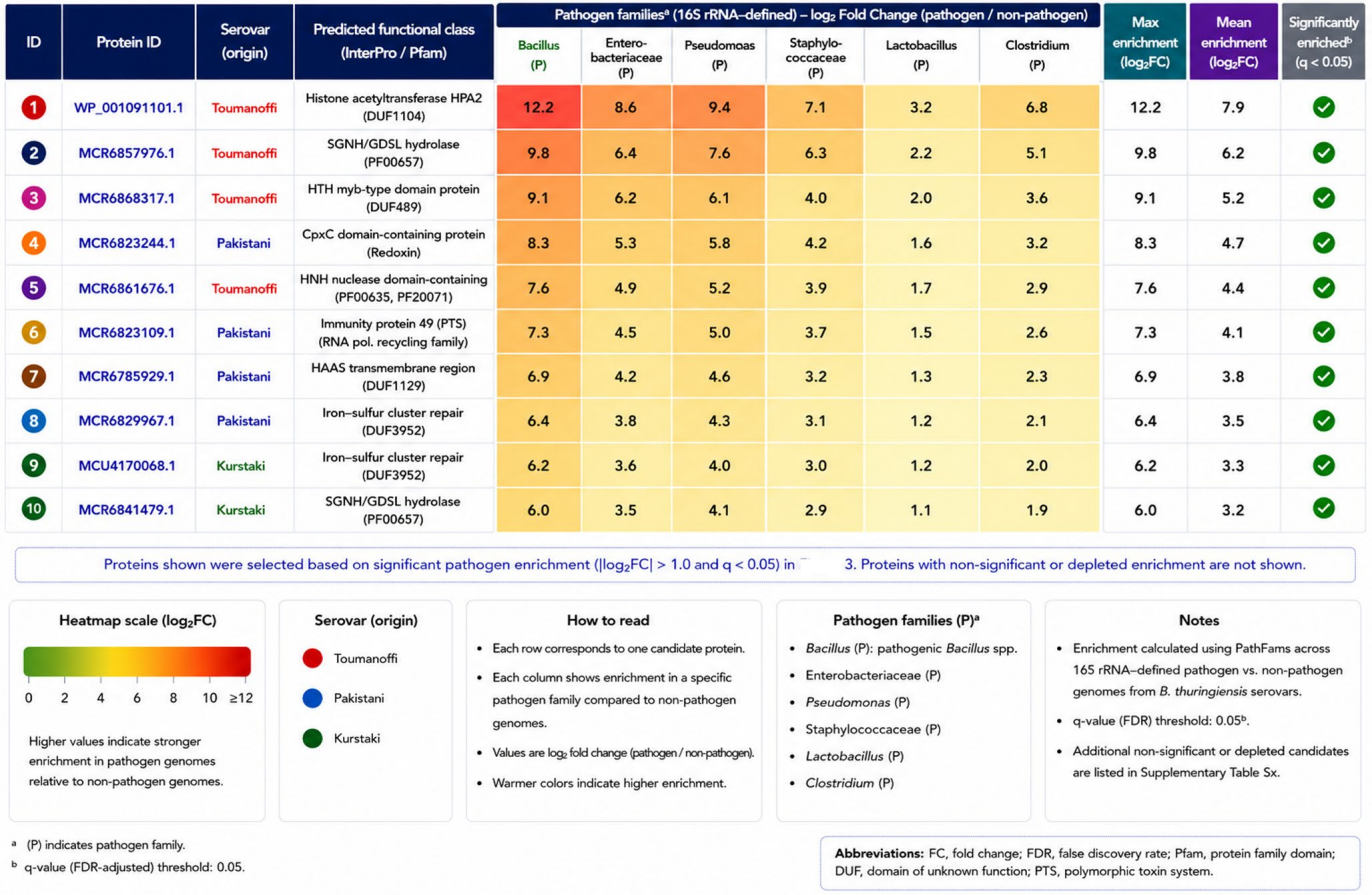
Comparative pathogen-enrichment profiles of significantly enriched hypothetical proteins across *Bacillus thuringiensis* serovars. Heatmap showing relative pathogen enrichment (log₂ fold change; pathogen vs. non-pathogen genomes) for the significantly enriched candidate proteins identified by PathFams analysis. Rows correspond to prioritized proteins, whereas columns represent major pathogen-associated bacterial groups defined using 16 S rRNA-based classification. Warmer colors indicate stronger enrichment in pathogen-associated genomes. The table additionally summarizes serovar origin, predicted functional class, maximum and mean enrichment values, and statistical significance (FDR-adjusted q < 0.05). Only proteins meeting the established enrichment thresholds in Fig. 3 are shown. Non-significant or depleted candidates are reported in Supplementary Table Sx

PathFams enrichment analyses identified a restricted subset of hypothetical proteins simultaneously exceeding the established significance thresholds (log₂FC > 1 and FDR-adjusted Q-value < 0.05), reinforcing the selectivity of the prioritization workflow. Most proteins remained below enrichment cutoffs, whereas only a limited group displayed both elevated fold-change values and strong statistical support (Figs. 3 and 4).

SGNH/GDSL hydrolases and iron–sulfur cluster repair proteins consistently ranked among the most significantly enriched candidates, exhibiting high fold-change values and low Q-values across independent serovars. Several enriched proteins were recurrently identified after redundancy reduction, supporting conservation of pathogen-associated functional signatures within the *Bacillus cereus* group.

Comparative enrichment profiles of the prioritized proteins are presented in Fig. 3, while the global enrichment distribution and significance landscape are illustrated in Fig. 4. Virulence-associated prediction outputs and enrichment metrics are additionally summarized in Supplementary Table S4.

### Structural prioritization of non-redundant candidates

Structural prioritization was performed after completion of functional annotation, enrichment analysis, and redundancy reduction. The resulting set of 11 non-redundant proteins was comparatively evaluated according to localization profiles, topology-associated features, sequence complexity, hydropathy, physicochemical stability, and DockingScore values.

Application of the DockingScore framework prioritized six proteins for downstream structural analyses: WP_001091101.1, MCR6868317.1, MCR6857976.1, MCR6823244.1, MCR6861676.1, and MCR6885802.1. These proteins exhibited favorable combinations of pathogen-enrichment support, localization consistency, reduced topological complexity, and higher structural-prioritization scores.

Most structurally prioritized proteins lacked transmembrane helices and displayed localization profiles compatible with soluble or extracellular conformations, facilitating downstream structural analyses. Notably, proteins presenting stronger pathogen-enrichment signals generally also exhibited higher DockingScore values, indicating concordance between enrichment-based prioritization and structural tractability.

**Fig. 5:**
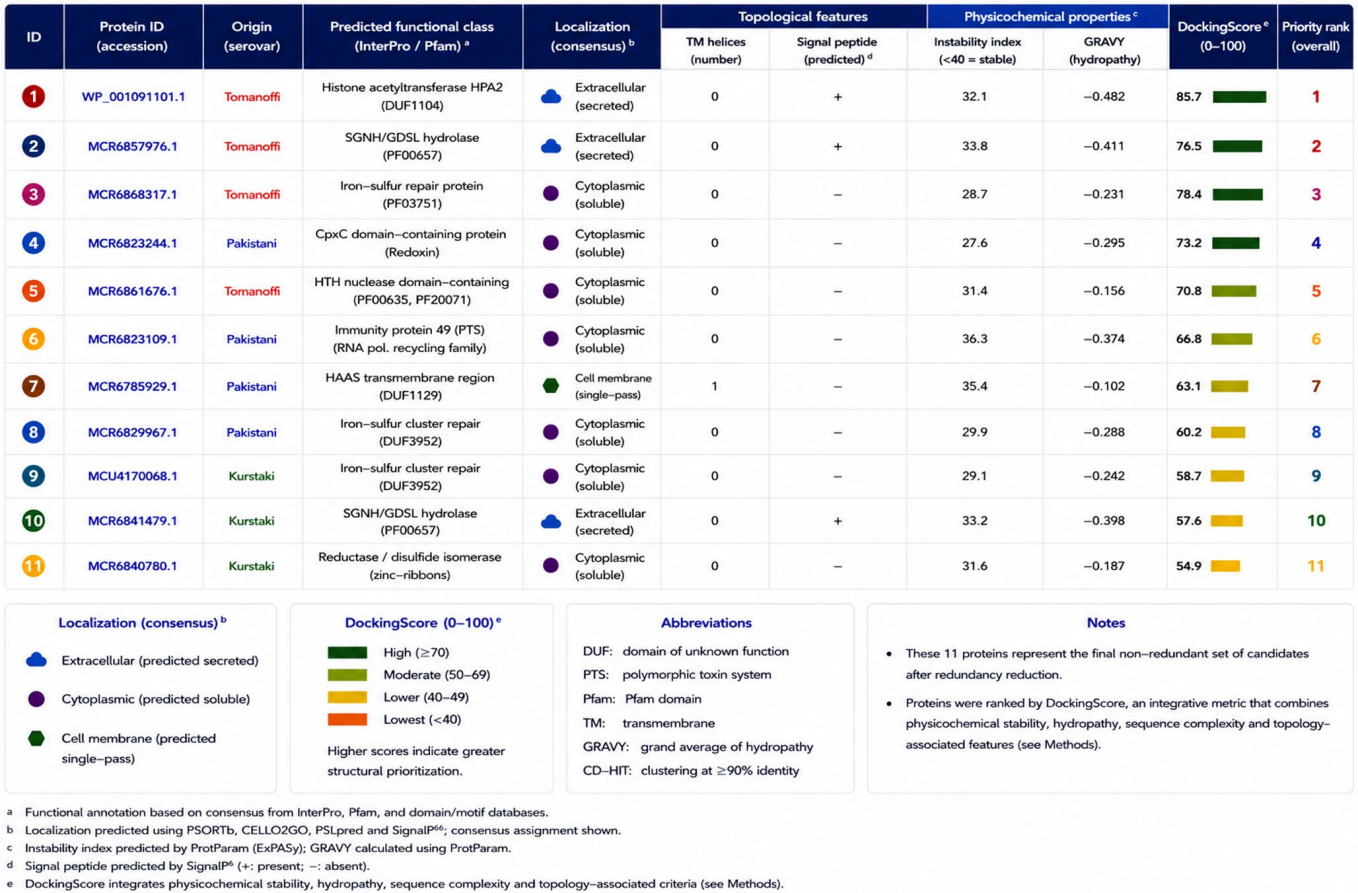
Integrated structural prioritization of the 11 non-redundant hypothetical proteins identified across *Bacillus thuringiensis* serovars. Candidates retained after redundancy reduction (CD-HIT, ≥90% sequence identity) were comparatively evaluated according to predicted functional class, subcellular localization, transmembrane topology, secretion signals, physicochemical stability, hydropathy, and integrative DockingScore values. Localization consensus was inferred using multiple prediction tools, whereas topology-associated features included transmembrane helices and signal peptide prediction. DockingScore values integrate physicochemical stability, hydropathy, sequence complexity, and topology-associated criteria to estimate structural suitability for downstream analyses. Higher scores indicate increased prioritization for comparative structural and docking-based evaluation

Comparative DockingScore rankings and integrative prioritization metrics are presented in Fig. 5, whereas detailed prioritization criteria are summarized in Supplementary Table S1.

### Comparative docking analyses

Comparative molecular docking analyses were performed for the six structurally prioritized proteins using functionally compatible ligand sets selected according to predicted functional classes. Predicted binding energies ranged from − 2.4 to − 5.9 kcal mol⁻¹, indicating variable interaction profiles among the selected candidates.

**Fig. 6:**
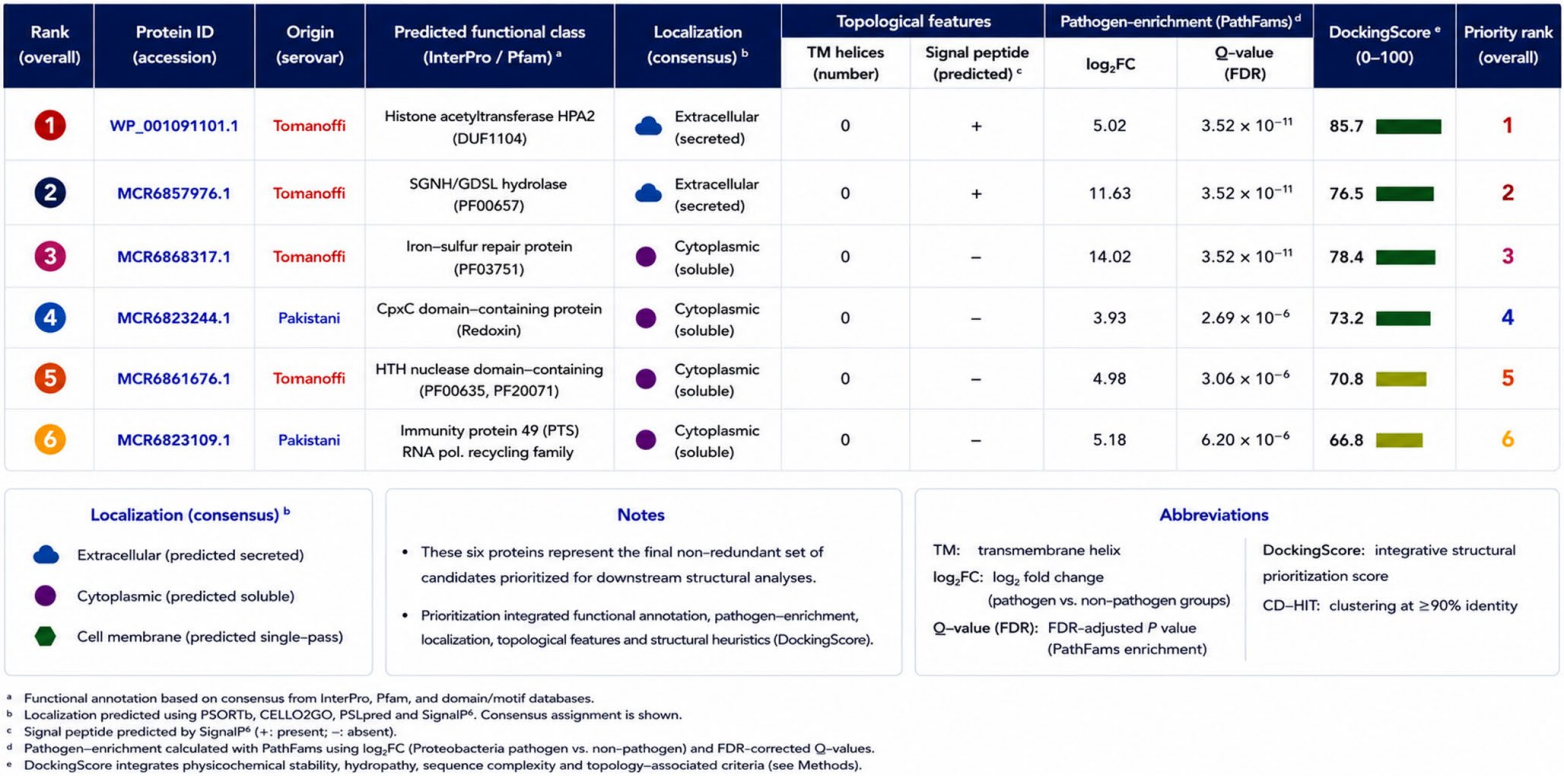
Final set of non-redundant hypothetical proteins prioritized for downstream structural analyses in *Bacillus thuringiensis* serovars. Six proteins were retained after integrative filtering based on consensus functional annotation, pathogen-enrichment analysis, subcellular localization profiling, topology-associated features, and structural prioritization criteria. The table summarizes predicted functional classes, localization profiles, transmembrane topology, secretion signals, pathogen-enrichment metrics (log₂FC and FDR-adjusted q-values), and integrative DockingScore values. Candidates with higher DockingScore values were prioritized for comparative docking and downstream structural analyses due to their favorable physicochemical and topological profiles

The final candidate set integrated pathogen-enrichment support, localization consistency, topological simplicity, and comparative structural suitability metrics derived from the DockingScore framework (Fig. 6). SGNH/GDSL hydrolases and HNH-domain proteins ranked among the most structurally promising candidates, combining favorable enrichment signatures with comparatively stable docking-energy distributions. Independent docking reruns generated consistent ranking patterns across simulations, supporting the reproducibility of the comparative prioritization framework.

The final candidate set integrated pathogen-enrichment support, consensus localization profiles, topology-associated simplicity, physicochemical tractability, and comparative docking-readiness metrics. Docking analyses were interpreted exclusively as comparative indicators of structural plausibility and ligand compatibility and should not be considered direct evidence of biochemical activity or functional validation.

The final set of structurally prioritized candidates is summarized in Fig. 6.

## Discussion

The systematic analysis of hypothetical proteins across multiple *Bacillus thuringiensis* serovars supports the usefulness of integrative bioinformatics frameworks for reducing functional uncertainty and prioritizing biologically plausible candidates. By combining consensus annotation, pathogen-enrichment analysis, redundancy reduction, localization profiling, physicochemical filtering, and structural prioritization, a large and heterogeneous set of uncharacterized proteins was progressively refined into a restricted subset supported by convergent computational evidence. Notably, the reduction from 2,052 initial hypothetical proteins to 11 non-redundant prioritized candidates highlights the selectivity and stringency of the workflow.

These findings reinforce the hypothesis that integrative computational approaches can improve the characterization of hypothetical proteins when compared with single-method annotation strategies. Previous studies have shown that hypothetical proteins are frequently associated with essential biological processes, including stress response, adaptive regulation, and antimicrobial resistance, emphasizing their functional relevance in microbial systems [16]. In *B. thuringiensis*, comparative genomic analyses have revealed extensive accessory genomic diversity and ecological versatility, supporting its characterization as a multifunctional bacterium with complex environmental interactions [2]. Within this context, the enrichment of oxidoreductases, hydrolases, regulatory proteins, and envelope-associated factors identified here is consistent with the possibility that subsets of hypothetical proteins contribute to adaptive and stress-associated processes complementary to the canonical Cry/Cyt toxin repertoire.

Despite the high sequence conservation observed among members of the *Bacillus cereus* group, several homologous proteins identified through BLASTp analyses remained annotated as hypothetical proteins in public databases. This observation highlights the persistence of functional uncertainty even among highly conserved bacterial proteins and further supports the need for systematic annotation frameworks capable of recovering biologically meaningful signals from poorly characterized genomic regions.

Previous studies have shown that accessory genomic regions contribute substantially to functional diversity and ecological adaptation within the *Bacillus cereus* group [2, 5]. Although genomic localization was not directly investigated in the present study, the enrichment of stress-associated and envelope-related proteins observed here is compatible with the hypothesis that subsets of hypothetical proteins may participate in adaptive processes linked to environmental specialization, persistence, and host-associated interactions.

Functional and mechanistic implications.

Among the prioritized candidates, SGNH/GDSL hydrolases and iron–sulfur (Fe–S) cluster repair proteins emerged as some of the most recurrent and significantly enriched functional signatures. SGNH/GDSL hydrolases were consistently identified across all analyzed serovars and displayed highly significant enrichment profiles. In Gram-positive bacteria, proteins belonging to this family have been associated with peptidoglycan remodeling, lipid modification, and envelope maintenance processes potentially related to environmental adaptation and stress tolerance [7].

Similarly, Fe–S cluster repair proteins exhibited some of the highest fold-change values observed in the dataset, supporting potential association with oxidative and nitrosative stress adaptation. Fe–S repair systems contribute to redox homeostasis and preservation of enzymatic activity under hostile environmental conditions [8]. The recurrence of these proteins across independent serovars after redundancy reduction suggests the existence of conserved adaptive signatures within the *Bacillus cereus* group.

Complementing these intracellular mechanisms, the enrichment of membrane-associated proteins, including DoxX-like proteins, HAAS-domain proteins, FtsH-associated proteins, and disulfide oxidoreductases, is consistent with potential envelope-associated stress-response and protein quality-control processes. Disulfide oxidoreductases are known to participate in oxidative folding pathways involved in stabilization and maturation of secreted or surface-associated proteins [9–11]. The coexistence of cytoplasmic regulators, membrane-associated proteins, and extracellular hydrolases therefore suggests coordinated adaptive strategies involving environmental sensing, envelope remodeling, intracellular regulation, and stress resilience.

Structural and physicochemical prioritization analyses further supported these functional interpretations. Previous studies have demonstrated that structural information and physicochemical profiling can provide complementary insights into protein function, particularly when sequence similarity alone is insufficient for robust annotation [12]. In the present study, comparative docking analyses were used exclusively as supportive prioritization tools rather than definitive evidence of biochemical activity. Proteins presenting higher DockingScore values generally exhibited more consistent docking-energy distributions and lower variability across ligand sets, supporting concordance between physicochemical prioritization and comparative docking profiles.

The comparatively narrow interaction-energy distributions observed for several prioritized proteins suggest internally consistent interaction patterns across docking simulations. Nevertheless, molecular docking approaches remain inherently limited by simplified energetic models and scoring-function inaccuracies [47, 48]. Accordingly, docking outputs were interpreted exclusively as comparative indicators of structural plausibility and ligand compatibility rather than direct evidence of molecular function.

### Interbacterial competition and regulatory dynamics

Additional prioritized candidates, including HNH-domain nucleases and helix–turn–helix (HTH) Myb-type regulators, suggest broader ecological and regulatory roles. HNH nucleases are commonly associated with DNA cleavage systems, polymorphic toxins, and interbacterial competition mechanisms, whereas HTH regulators are frequently involved in transcriptional regulation and environmental adaptation. Previous studies have demonstrated that hypothetical proteins may participate in poorly characterized interaction networks associated with microbial competition, signaling, and adaptive regulation [49].

Comparative docking analyses of HNH-domain proteins were consistent with predicted nucleic-acid interaction potential inferred from conserved domain annotations. However, these observations remain computational predictions and should therefore be interpreted as hypothesis-generating evidence rather than confirmatory functional assignments.

### Integrative perspective and biological relevance

Collectively, the integration of consensus annotation, pathogen-enrichment analysis, localization profiling, redundancy reduction, physicochemical filtering, and structural prioritization enabled the identification of a restricted subset of hypothetical proteins supported by internally consistent computational evidence. The convergence observed among enrichment metrics, recurrent domain architectures, localization profiles, and structural-prioritization outputs supports the hypothesis that hypothetical proteins contain recoverable functional signals that can be systematically extracted through integrative computational frameworks.

The resulting functional landscape suggests that the prioritized proteins may participate in complementary adaptive processes involving stress response, envelope maintenance, redox homeostasis, regulatory control, and environmental adaptation. In *B. thuringiensis*, these auxiliary systems may contribute to persistence across diverse ecological niches, including plant-associated and insect-associated environments relevant to biological pest control. Importantly, all biological interpretations presented herein are based exclusively on computational inference and should therefore be regarded as prioritized biological hypotheses rather than definitive functional assignments.

### Limitations and perspectives

A primary limitation of this study is its reliance on computational analyses. Although integration of multiple independent tools, conservative consensus filtering, explicit enrichment thresholds, redundancy reduction, and independent reruns of docking procedures improved internal consistency, these approaches cannot substitute for experimental validation. Previous studies have demonstrated that combining docking analyses with complementary structural approaches, including molecular dynamics simulations, may improve the reliability of interaction predictions and structural assessments [50].

Future investigations should focus on experimental validation of prioritized candidates through transcriptomic analyses, mutational assays, biochemical characterization, and phenotypic evaluation under stress-associated conditions. Additional analyses involving genomic context, operon organization, plasmid localization, and gene-neighborhood conservation may further refine functional interpretation and improve biological relevance.

Collectively, these approaches may contribute to reducing the persistent annotation gap associated with hypothetical proteins and support the development of more comprehensive functional maps for the Bacillus cereus group.

## Conclusions

This study demonstrates that a consensus-based and reproducible integrative bioinformatics workflow can effectively reduce the functional uncertainty associated with hypothetical proteins in *Bacillus thuringiensis*. By combining domain validation, homology inference, virulence-associated prediction, pathogen-enrichment analysis, subcellular localization profiling, and structural prioritization, a large and heterogeneous dataset of uncharacterized proteins was progressively refined into a focused subset of biologically plausible candidates across three *B. thuringiensis* serovars.

Sequential filtering reduced an initial dataset of 2,052 hypothetical proteins and DUF-containing sequences to 11 non-redundant prioritized protein entities supported by convergent computational evidence. Among these, SGNH/GDSL hydrolases, iron–sulfur cluster repair proteins, regulatory proteins, and envelope-associated factors emerged as recurrent functional signatures across independent serovars, suggesting the existence of conserved adaptive systems within the *Bacillus cereus* group. These findings support the view that adaptive traits in *B. thuringiensis* extend beyond the canonical Cry/Cyt toxin repertoire and may involve a broader functional landscape embedded within the hypothetical-protein fraction of the genome.T.

Integration of physicochemical filtering and comparative structural prioritization further enabled the identification of six candidates displaying favorable computational profiles for downstream structural and functional analyses. These proteins represent promising targets for future experimental characterization and may support comparative functional genomics and biotechnological investigations involving *Bacillus* spp. and related bacterial systems.

Although based exclusively on computational analyses, the proposed workflow provides a scalable and transferable framework for narrowing the experimental search space and supporting hypothesis-driven validation. Overall, this strategy contributes to bridging the gap between genomic information and functional interpretation, facilitating the systematic characterization of hypothetical proteins in *Bacillus* and related bacterial genomes.

## Supplementary Information

Supplementary Material 1.

Supplementary Material 2.

Supplementary Material 3.

Supplementary Material 4.

Supplementary Material 5.

## Data Availability

All data generated or analyzed during this study are included in this published article and its supplementary information files.
