# Supplementary material for: An integrative bioinformatics framework for functional annotation and prioritization of hypothetical proteins in Bacillus thuringiensis relevant to biological pest control
Source: Braz J Microbiol. 2026 Jun 10;57(1):170. doi: 10.1007/s42770-026-01985-x (PMC13253909; doi:10.1007/s42770-026-01985-x)
Supplement: Supplementary file 1 — Supplementary Material 1. [file 42770_2026_1985_MOESM1_ESM.pdf]

Supplementary Table S1. Criteria used for prioritization of hypothetical proteins (HPs) and scoring scheme.

Criterion	Measurement / Tool	Optimal range (score kept)	Penalization applied	Reference(s)
Sequence length	ProtParam	120–800 aa	<120 or >800 aa (–10)	Gasteiger et al., 2005
Ambiguous residues	ProtParam	≤1%	>1% (discarded)	Rehman et al., 2025
Instability index	ProtParam	<40	≥40 (–10)	Guruprasad et al., 1990
Aliphatic index	ProtParam	Recorded only	—	Ikai, 1980
GRAVY (hydropathy)	ProtParam / Kyte–Doolittle	<0	>0 (–15)	Kyte & Doolittle, 1982
Isoelectric point (pI)	ProtParam	Recorded only	—	Bjellqvist et al., 1993
Transmembrane helices	TMHMM / DeepTMHMM / CCTOP	None	≥1 helix (discarded)	Krogh et al., 2001
Signal peptide	SignalP 6.0	Absent	Present (–15)	Teufel et al., 2022
Low-complexity fraction	Shannon entropy (12 aa windows)	<10%	≥10% (–10)	Uversky, 2019
DockingScore	Docking pipeline	≥70	<70 (discarded)	This study
