# Supplementary material for: An integrative bioinformatics framework for functional annotation and prioritization of hypothetical proteins in Bacillus thuringiensis relevant to biological pest control
Source: Braz J Microbiol. 2026 Jun 10;57(1):170. doi: 10.1007/s42770-026-01985-x (PMC13253909; doi:10.1007/s42770-026-01985-x)
Supplement: Supplementary file 2 — Supplementary Material 2. [file 42770_2026_1985_MOESM2_ESM.pdf]

Supplementary Table S3. Consensus functional annotation of candidate hypothetical proteins supported by at least three independent bioinformatics tools.

Protein ID	Description	HHPred Annotation	COMER Annotation	SUPERFAMILY Annotation	Motif/Domain Annotation
MCR6838482.1	Cytochrome b561/Ni-hydrogenase domain protein	Metalloreductase	Electron transfer, cofactor-binding	Cytochrome c oxidase assembly	
MCR6838517.1	Pectate lyase superfamily protein	Polygalacturonidase / Pectinase	Pectate lyase superfamily	Pectin lyase-like	Pectate_lyase_3
MCR6838645.1	YfjM / YaaA protein	Cytoplasmic iron regulator	Iron homeostasis protein	Peroxide stress protein YaaA	
MCR6839227.1	N-acetyltransferase protein	Acyl-homoserine-lactone synthase	Acyl transferase	Acyl-CoA N-acyltransferase	
MCR6840780.1	Reductase / disulfide isomerase (YcnL)	Nucleic acid-binding protein	Zn-ribbon domain	ARM repeat zinc ribbon	Zinc-ribbon C4-type
MCR6840862.1	CopG ribbon-helix-helix protein	DNA-binding protein	Zinc finger protein	Ribbon-helix-helix domain	
MCR6841065.1	CopG transcriptional regulator	DNA-binding regulator	Ribbon-helix-helix	Transcriptional regulator	XPG-like domain
MCR6841068.1	LAGLIDADG endonuclease	DNA-binding regulator	LAGLIDADG	Homing endonuclease	LAGLIDADG domain
MCR6841174.1	AbrB transcriptional regulator	DNA-binding regulator	Sporulation regulator	RNA recognition motif	
MCR6841256.1	DNA polymerase	DNA polymerase II	DNA-binding enzyme	DNA polymerase family B	
MCR6841265.1	Zona occludens toxin domain protein	ATPase-like protein	Nitrogenase-like domain	Zona occludens toxin	
MCR6841273.1	HTH transcriptional regulator	DNA-binding protein	Regulatory protein	Homeodomain-like	HTH domain
MCR6841479.1	SGNH/GDSL hydrolase	SGNH hydrolase	SGNH domain protein	Hydrolase superfamily	SGNH domain
MCR6841644.1	CYTH domain protein	Triphosphate tunnel enzyme	CYTH-like enzyme	CYTH domain	

MCR6841741.1	Pectate lyase protein	Galacturonidase	Pectin lyase-like	Pectate lyase	Pectate_lyase_3
MCR6843927.1	CYTH domain protein	Triphosphate tunnel enzyme	CYTH-like enzyme	CYTH-like phosphatase	
MCR6844143.1	Exosporium protein D	Spore-associated protein	Cupredoxin-like	Endospore protein	
MCR6844656.1	Flp pilus assembly protein (CpaE)	ATPase CpaE	Nitrogenase-like protein	Pilus assembly ATPase	
MCR6844675.1	Type VII secretion protein EssB	Membrane protein	ESX secretion system	YukC family protein	

Note: Functional annotations were derived from consensus predictions across multiple independent bioinformatics tools (HHPred, COMER, SUPERFAMILY, and motif/domain databases). Only proteins supported by at least three methods were retained.
