# Supplementary material for: An integrative bioinformatics framework for functional annotation and prioritization of hypothetical proteins in Bacillus thuringiensis relevant to biological pest control
Source: Braz J Microbiol. 2026 Jun 10;57(1):170. doi: 10.1007/s42770-026-01985-x (PMC13253909; doi:10.1007/s42770-026-01985-x)
Supplement: Supplementary file 3 — Supplementary Material 3. [file 42770_2026_1985_MOESM3_ESM.pdf]

Supplementary Table S4. Virulence prediction and enrichment analysis of candidate hypothetical proteins based on integrative bioinformatics tools.

Protein ID	Description	VirulencePred	BTXpred	VICM	PathFams	E-value	Fold change	Q-value	Domain
MCR6838482.1	Cytochrome b561 protein	Virulent	Bacterial toxin	Metabolism	path -	0.00018	0.872	1	Ferric reductase-like
MCR6838517.1	Pectate lyase protein	Virulent	Bacterial toxin	Cellular process	no				
MCR6838645.1	YfjM protein	Virulent	Non-toxin	Metabolism	path ++	4.9e-8	1.316	3.37e-6	Peroxide stress protein
MCR6839227.1	N-acetyltransferase	Virulent	Bacterial toxin	Metabolism	path -	0.000017	0.944	1	GNAT family
MCR6840780.1	YcnL protein	Virulent	Bacterial toxin	Metabolism	path ++	0.000048	5.027	1.26e-10	Zinc-ribbon
MCR6840862.1	CopG protein	Virulent	Bacterial toxin	Metabolism	path -	0.00033	0.875	1	RHH domain
MCR6841065.1	CopG regulator	Virulent	Bacterial toxin	Information	no				
MCR6841068.1	LAGLIDADG protein	Virulent	Bacterial toxin	Information	path +	0.0000056	1.171	0.113	LAGLIDADG
MCR6841174.1	AbrB regulator	Virulent	Bacterial toxin	Cellular	no				
MCR6841256.1	DNA polymerase	Bacterial toxin		Information	no				
MCR6841265.1	Zona occludens toxin	Virulent	Bacterial toxin	Metabolism	path -	0.0000083	1.009	0.262	AAA ATPase
MCR6841273.1	HTH regulator	Virulent	Bacterial toxin	Metabolism	path +	0.000031	1.038	1	HTH
MCR6841479.1	SGNH hydrolase	Bacterial toxin		Metabolism	path ++	5.3e-8	3.162	3.52e-11	DltD
MCR6841644.1	CYTH protein	Virulent	Bacterial toxin		no				
MCR6841741.1	Pectate lyase	Bacterial		Virulence	no				

		toxin							
MCR6843927.1	CYTH protein	Virulent	Bacterial toxin	Information	no				
MCR6844143.1	Exosporium protein	Virulent	Bacterial toxin	Information	no				
MCR6844656.1	CpaE protein	Bacterial toxin		Metabolism	no				
MCR6844675.1	EssB protein	Bacterial toxin		Metabolism	no				

Note: Virulence predictions were obtained using VirulencePred, BTXpred, and VICM. PathFams analysis was applied using enrichment criteria (fold change > 1 and Q < 0.05). Only high-confidence predictions were retained.
