# Supplementary material for: An integrative bioinformatics framework for functional annotation and prioritization of hypothetical proteins in Bacillus thuringiensis relevant to biological pest control
Source: Braz J Microbiol. 2026 Jun 10;57(1):170. doi: 10.1007/s42770-026-01985-x (PMC13253909; doi:10.1007/s42770-026-01985-x)
Supplement: Supplementary file 5 — Supplementary Material 5. [file 42770_2026_1985_MOESM5_ESM.pdf]

Supplementary Table **S**2. Blastp search results for similar sequences in the non-redundant (nr) protein database**.**

BLASTp similarity search results against the NCBI non-redundant (nr) database showing positive matches with high identity and query coverage for the analyzed sequences, predominantly corresponding to proteins annotated as hypothetical across multiple *Bacillus* species.

sequence	organism	Max Score	Total Score	Query Cover	E value	Per. Ident	Acc. Len	Accession
WP_001091101.1	Bacillus cereus group	513	513	1	0.0	97.27%	256	WP_097938737.1
MCU4173096.1 DUF4064	Bacillus cereus group	217	217	1	1e-70	100.00%	115	WP_001255046.
MCU4170068.1 DUF3952	Bacillus cereus group	533	533	1	0.0	100.00%	265	WP_003286219.1
MCR6868317.1	Bacillus cereus group	461	461	1	2e-163	100.00%	229	WP_000528552.1
MCR6864009.1	Bacillus cereus group	308	308	1	3e-105	100.00%	151	WP_000066449.1
MCR6861676.1	Bacillus cereus group	912	912	0.78	0.0	99.11%	447	HDR8361147.1
MCR6857976.1	Bacillus thuringiensis serovar toumanoffi	786	786	1	0.0	100.00%	400	OTZ56179.1
MCR6841479.1	Bacillus	782	782	1	0.0	100.00%	382	WP_000664253.1
MCR6840780.1	Bacillus	223	223	1	4e-73	100.00%	113	WP_000658667.1
MCR6830118.1	Bacillus cereus group	781	781	1	0.0	100.00%	382	WP_153588931.1
MCR6829967.1	Bacillus cereus group	534	534	1	0.0	100.00%	265	WP_153589096.1
MCR6823244.1	Bacillus cereus group	464	464	1	6e-165	100.00%	224	WP_153588306.1
MCR6823109.1	Bacillus cereus group	503	503	1	1e-179	100.00%	242	WP_153588268.1
MCR6785929.1	Bacillus cereus group	493	493	1	5e-175	100.00%	253	WP_088066409.1
